# Disparities in Internet Access and COVID-19 Vaccination in New York City

**DOI:** 10.5888/pcd18.210143

**Published:** 2021-08-26

**Authors:** Isaac H. Michaels, Sylvia J. Pirani, Alvaro Carrascal

**Affiliations:** 1 Department of Epidemiology and Biostatistics, University at Albany School of Public Health, Rensselaer, New York; 2 HRSA Funded Region 2 Public Health Training Center, Columbia University Mailman School of Public Health, Department of Sociomedical Sciences, New York, New York

**Figure:**
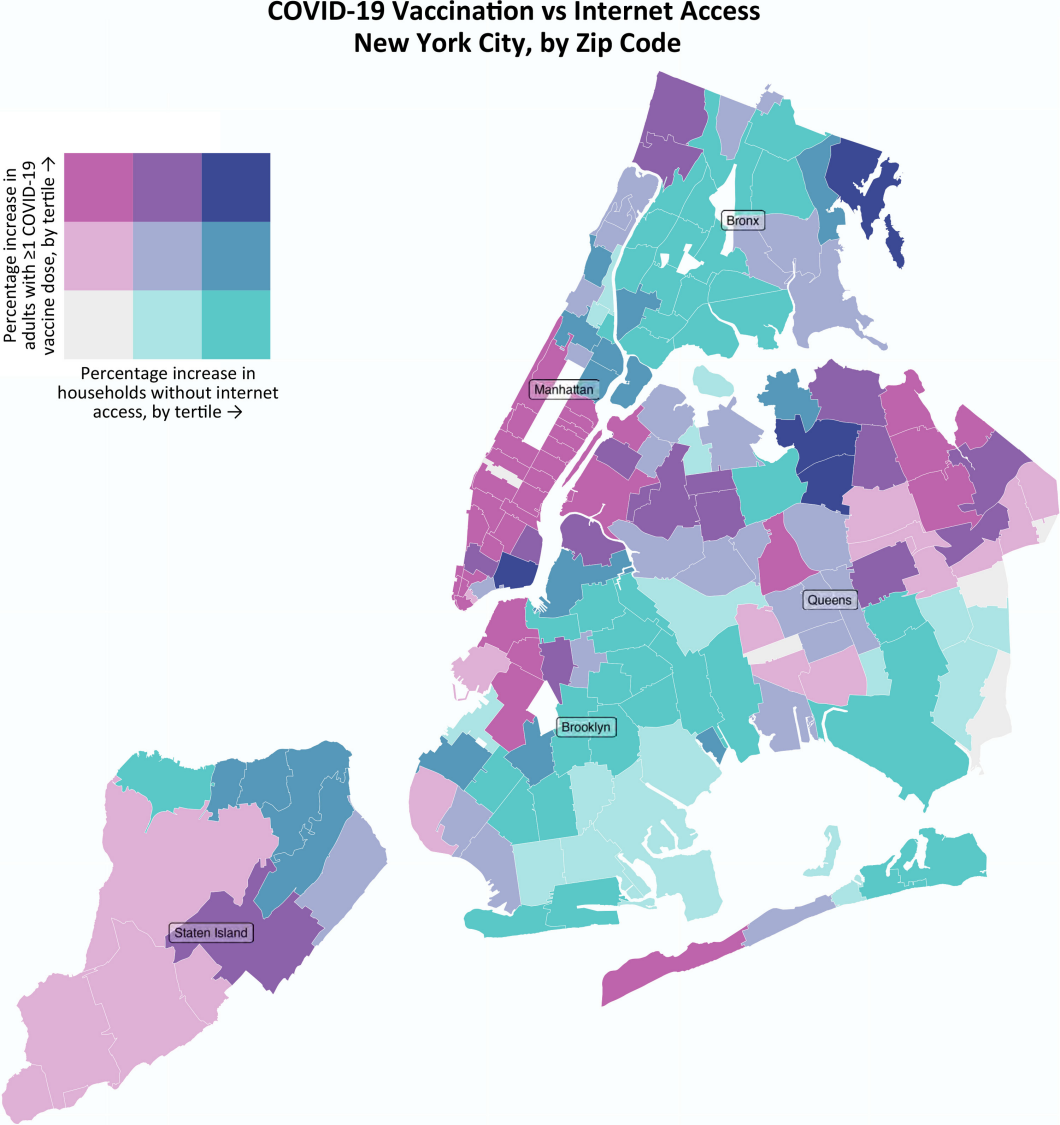
A bivariate choropleth map that visualizes zip code–level data on household internet access and COVID-19 vaccination in New York City. The map identifies zip codes where the greatest disparities exist. Most disparities are in the Bronx and Brooklyn. This information could be used to place appointment-free vaccination sites in the short term and augment digital education and broadband internet access in the long term. Data on internet access were retrieved from NYC Open Data on April 7, 2021. Vaccination data were retrieved from the New York City Department of Health and Mental Hygiene on April 7, 2021.

**Table:** 

Modified Zip Code Tabulation Area	Percentage of Households Without Internet Access	Percentage of Adults With ≥1 COVID-19 Vaccine Dose	Neighborhood
10001	9.1	44.9	Chelsea/NoMad/West Chelsea
10002	30.9	47.6	Chinatown/Lower East Side
10003	8.0	44.7	East Village/Gramercy/Greenwich Village
10004	0.8	64.9	Financial District
10005	1.2	42.6	Financial District
10006	1.4	47.2	Financial District
10007	9.3	62.2	TriBeCa
10009	17.2	44.5	Alphabet City/East Village/Stuyvesant Town-Cooper Village
10010	8.4	46.8	Flatiron/Gramercy/Kips Bay
10011	5.9	52.5	Chelsea
10012	11.2	45.2	Greenwich Village/SoHo
10013	15.5	52.2	Hudson Square/Little Italy/SoHo/TriBeCa
10014	6.1	50.1	Hudson Square/Meatpacking District/West Village
10016	7.7	51.7	Kips Bay/Murray Hill/NoMad
10017	3.9	53.1	East Midtown/Murray Hill
10018	5.6	33.3	Hell's Kitchen/Midtown Manhattan
10019	9.0	49.5	Hell's Kitchen/Midtown Manhattan
10021	5.8	58.0	Lenox Hill/Upper East Side
10022	7.0	59.9	East Midtown
10023	6.6	56.3	Lincoln Square
10024	7.2	57.5	Upper West Side
10025	11.5	51.7	Manhattan Valley/Morningside Heights/Upper West Side
10026	18.3	34.5	Central Harlem (South)
10027	19.6	34.0	Central Harlem (South)/Morningside Heights/West Harlem
10028	4.6	53.8	Upper East Side/Yorkville
10029	22.1	36.1	East Harlem
10030	18.4	28.5	Central Harlem (North)
10031	18.1	35.0	Hamilton Heights/West Harlem
10032	20.5	38.3	Washington Heights (South)
10033	17.0	35.8	Washington Heights (North)/Washington Heights (South)
10034	16.9	34.5	Inwood/Washington Heights (North)
10035	27.8	39.2	East Harlem
10036	11.4	50.3	Hell's Kitchen/Midtown Manhattan
10037	20.8	32.8	Central Harlem (North)/East Harlem
10038	16.7	42.1	Chinatown/Financial District
10039	18.0	27.9	Central Harlem (North)/Washington Heights (South)
10040	15.6	37.2	Washington Heights (North)
10044	7.7	47.3	Roosevelt Island
10065	6.9	60.1	Lenox Hill/Upper East Side
10069	3.9	53.3	Lincoln Square
10075	7.1	66.2	Lenox Hill/Upper East Side
10128	9.9	55.9	Upper East Side/Yorkville
10280	1.0	44.9	Battery Park City
10282	0.0	44.3	Battery Park City
10301	19.0	40.9	Silver Lake/St. George
10302	19.2	33.5	Elm Park
10303	20.8	31.8	Graniteville/Mariner's Harbor/Port Ivory
10304	22.5	39.9	New Dorp/Todt Hill
10305	16.9	40.7	Arrochar/Midland Beach/Shore Acres/South Beach Ocean Breeze
10306	14.9	44.5	Lighthouse Hill/Midland Beach/New Dorp/Oakwood
10307	12.2	36.0	Tottenville
10308	10.6	38.7	Great Kills
10309	10.8	38.2	Charleston/Prince's Bay/Woodrow
10310	23.4	42.1	Port Richmond/Randall Manor/West Brighton
10312	10.7	41.8	Annadale/Rossville
10314	11.7	40.6	Bloomfield/Freshkills Park
10451	27.6	34.9	Concourse/Melrose
10452	26.2	31.8	Concourse/Highbridge
10453	26.2	28.0	Morris Heights/Mount Hope/University Heights
10454	32.4	28.2	Mott Haven/Port Morris
10455	28.2	29.3	Mott Haven
10456	26.0	28.0	Claremont/Morrisania
10457	26.8	27.8	Belmont/Claremont/Mount Hope/Tremont
10458	20.9	26.9	Belmont/Fordham University/Kingsbridge
10459	28.1	29.6	Charlotte Gardens/Hunts Point
10460	28.5	27.6	Charlotte Gardens/Tremont/Van Nest/West Farms
10461	17.6	43.1	Morris Park/Pelham Bay/Westchester Square
10462	17.6	37.8	Parkchester/Pelham Parkway/Van Nest/Westchester Square
10463	15.6	43.6	Kingsbridge/Marble Hill/Riverdale/Spuyten Duyvil
10464	24.1	54.8	City Island
10465	15.2	38.8	Country Club/Throgs Neck
10466	19.3	25.7	Edenwald/Wakefield
10467	20.6	29.3	Allerton/Norwood/Pelham Parkway/Williamsbridge
10468	20.0	28.9	Fordham/Kingsbridge/University Heights
10469	19.0	31.2	Allerton/Baychester/Pelham Gardens/Williamsbridge
10470	17.7	34.1	Wakefield/Woodlawn
10471	13.8	51.1	Fieldston/North Riverdale/Riverdale
10472	24.3	28.4	Soundview
10473	20.4	32.3	Castle Hill/Clason Point/Soundview
10474	28.3	23.4	Hunts Point
10475	21.0	40.9	Co-op City/Edenwald
11004	12.5	43.4	Bellerose/Douglaston-Little Neck
11101	12.1	50.8	Astoria (South)/Long Island City/Sunnyside
11102	12.2	46.9	Astoria (North)
11103	13.2	40.3	Astoria (North)/Astoria (South)
11104	14.1	46.6	Sunnyside
11105	13.2	38.2	Ditmars Steinway
11106	13.9	43.9	Astoria (South)
11109	2.1	50.8	Long Island City
11201	9.6	53.1	Brooklyn Heights/DUMBO/Downtown Brooklyn
11203	21.5	27.8	East Flatbush (North)/East Flatbush (South)
11204	24.9	31.8	Bensonhurst/Mapleton
11205	21.8	31.3	Bedford-Stuyvesant (West)/Clinton Hill/Fort Greene
11206	26.8	30.0	Williamsburg (South)
11207	22.0	24.5	Cypress Hills/East New York
11208	19.2	25.2	Cypress Hills/East New York
11209	12.8	42.3	Bay Ridge/Fort Hamilton
11210	14.0	25.6	Flatlands/Midwood
11211	26.7	35.6	East Williamsburg/Williamsburg (North)/Williamsburg (South)
11212	27.0	24.5	Ocean Hill-Brownsville
11213	23.1	25.3	Crown Heights (East)
11214	17.0	33.7	Bath Beach/Bensonhurst/Gravesend
11215	6.8	50.5	Gowanus/Park Slope/Windsor Terrace
11216	17.8	37.7	Bedford-Stuyvesant (West)/Crown Heights (West)
11217	7.9	45.4	Boerum Hill/Park Slope
11218	18.7	37.8	Kensington/Windsor Terrace
11219	31.9	25.1	Borough Park
11220	22.2	36.4	Sunset Park
11221	21.5	31.9	Bedford-Stuyvesant (East)/Bushwick
11222	13.7	44.8	Greenpoint
11223	17.2	29.1	Gravesend/Homecrest
11224	26.5	27.6	Brighton Beach/Coney Island/Seagate
11225	20.4	33.3	Crown Heights (West)/Prospect Lefferts Gardens
11226	22.4	30.2	Flatbush/Prospect Lefferts Gardens
11228	13.9	38.2	Bath Beach/Dyker Heights
11229	15.2	32.7	Gerritsen Beach/Homecrest/Sheepshead Bay
11230	20.3	27.7	Midwood
11231	12.6	43.5	Carroll Gardens/Cobble Hill/Red Hook
11232	17.1	33.4	Sunset Park
11233	24.8	24.4	Bedford-Stuyvesant (East)/Ocean Hill-Brownsville
11234	13.8	30.8	Bergen Beach/Flatlands/Marine Park/Mill Basin
11235	19.7	31.8	Brighton Beach/Manhattan Beach/Sheepshead Bay
11236	16.1	24.7	Canarsie
11237	18.8	33.3	Bushwick/East Williamsburg
11238	13.0	49.8	Clinton Hill/Prospect Heights
11239	25.4	41.4	East New York
11354	24.7	46.5	Flushing/Murray Hill
11355	24.3	46.1	Flushing/Murray Hill/Queensboro Hill
11356	20.2	40.0	College Point
11357	13.8	46.1	Whitestone
11358	14.0	46.1	Auburndale/Murray Hill
11360	11.0	49.9	Bayside (North)
11361	10.4	46.9	Bayside (North)/Bayside (South)
11362	14.3	48.3	Douglaston-Little Neck
11363	12.8	51.7	Douglaston-Little Neck
11364	8.9	48.1	Bayside (South)/Oakland Gardens
11365	12.3	41.3	Auburndale/Fresh Meadows/Pomonok/Utopia
11366	8.9	41.6	Fresh Meadows/Hillcrest
11367	15.3	36.2	Kew Gardens Hills/Pomonok
11368	19.6	26.4	Corona/North Corona
11369	18.1	37.7	Airport/East Elmhurst
11370	14.8	33.3	Jackson Heights/Rikers Island
11372	17.1	45.5	Jackson Heights
11373	16.1	45.9	Elmhurst
11374	14.9	40.5	Rego Park
11375	11.6	44.4	Forest Hills
11377	15.1	43.5	Woodside
11378	13.2	35.2	Maspeth
11379	14.1	38.8	Middle Village
11385	17.0	31.9	Glendale/Ridgewood
11411	13.2	26.5	Cambria Heights
11412	16.6	25.5	St. Albans
11413	15.5	24.1	Laurelton/Rosedale
11414	14.0	35.0	Hamilton Beach/Howard Beach/Lindenwood
11415	13.8	40.0	Kew Gardens
11416	11.7	32.7	Ozone Park
11417	11.4	36.7	Ozone Park
11418	14.2	35.9	Richmond Hill
11419	13.4	35.0	Richmond Hill/South Ozone Park
11420	12.2	36.3	South Ozone Park
11421	12.3	34.1	Woodhaven
11422	11.5	24.5	Rosedale
11423	12.5	38.5	Hollis/Holliswood
11426	10.2	39.0	Bellerose
11427	13.2	48.7	Bellerose/Hollis Hills/Holliswood
11428	12.0	42.8	Queens Village
11429	12.2	26.2	Queens Village
11432	15.4	45.8	Hillcrest/Jamaica Estates/Jamaica Hills
11433	23.3	28.6	Jamaica
11434	22.8	25.5	Airport/South Jamaica/Springfield Gardens/St. Albans
11435	15.8	34.7	Briarwood/Jamaica
11436	16.2	27.8	South Jamaica/South Ozone Park
11691	21.3	21.9	Edgemere/Far Rockaway
11692	19.2	26.9	Arverne/Edgemere
11693	18.5	32.2	Arverne/Broad Channel
11694	15.6	43.1	Belle Harbor-Neponsit/Rockaway Park
11697	12.2	75.0	Breezy Point

## Background

Although COVID-19 is a communicable disease, for many people it could also be a chronic disease (1). In New York City, COVID-19 has had a greater effect on older populations, people living in the outer boroughs of the city’s 5 boroughs (eg, the Bronx, Brooklyn, and Queens, compared with Manhattan), people living in poverty, and racial/ethnic minority populations (2). The availability of a COVID-19 vaccine can lessen the effect of the disease on these populations. As of this writing (April 15, 2021), people in New York City had to schedule an appointment to be vaccinated (3), with certain exceptions (4). Many New Yorkers who wanted to be vaccinated were not able to secure appointments, in part because the supply of COVID-19 vaccine was limited (5). Many public health and health care providers use online systems to schedule appointments; as a result, lack of internet access has been suggested as a potential barrier to vaccination (6). We created a map to visualize the community-level distribution of household internet access and COVID-19 vaccination in New York City.

## Data and Methods

We obtained estimates of the percentage of households lacking internet access, by zip code, from NYC Open Data (7). Data on internet access were collected by the 2018 5-year American Community Survey. According to NYC Open Data, the American Community Survey frames this question as having “No access to the internet at this house, apartment, or mobile home.”

We obtained data on zip code–level percentages of adult residents who received at least 1 COVID-19 vaccine dose from the New York City Department of Health and Mental Hygiene website (8). The vaccination data were obtained from the Citywide Immunization Registry. According to the source data table, “People with at least one dose have received at least one dose of two-dose vaccine series or a single dose shot.”

We calculated via simple linear regression the association between the percentage of households without internet access and the percentage of adult residents with at least 1 COVID-19 vaccine dose. Internet access and vaccination data were available for all populated (n = 177) modified zip code tabulation areas in New York City (9). We then classified zip codes into 3 internet-access quantiles (tertiles) and 3 COVID-19 vaccination quantiles and visualized them via a bivariate choropleth map (10). We developed the map in R version 4.0.4 (R Foundation for Statistical Computing) by using the tidyverse (11), sf (12), biscale (13), and cowplot (14) packages. All data were retrieved on April 7, 2021.

## Highlights

Among zip codes in New York City, the median percentage of households without internet access was 15.5% (SD, 6.7%), ranging from 0% (zip code 10282, Battery Park City, Manhattan) to 32.4% (zip code 10454, Mott Haven/Port Morris, the Bronx). The median percentage of adults that received at least 1 dose of COVID-19 vaccine was 38.2% (SD, 10.0%), ranging from 21.9% (zip code 11691, Edgemere/Far Rockaway, Queens) to 75.0% (zip code 11697, Breezy Point, Queens). In the simple linear regression model, the percentage of households without internet access was negatively associated with the percentage of adult residents who received at least 1 dose of COVID-19 vaccine (β = −0.92; 95 CI, −1.09 to −0.75; intercept = 53.7%; *P* < .001, adjusted *R* ^2^ = 0.38) (Figure). Most disparities were in the Bronx and Brooklyn.

**Figure:**
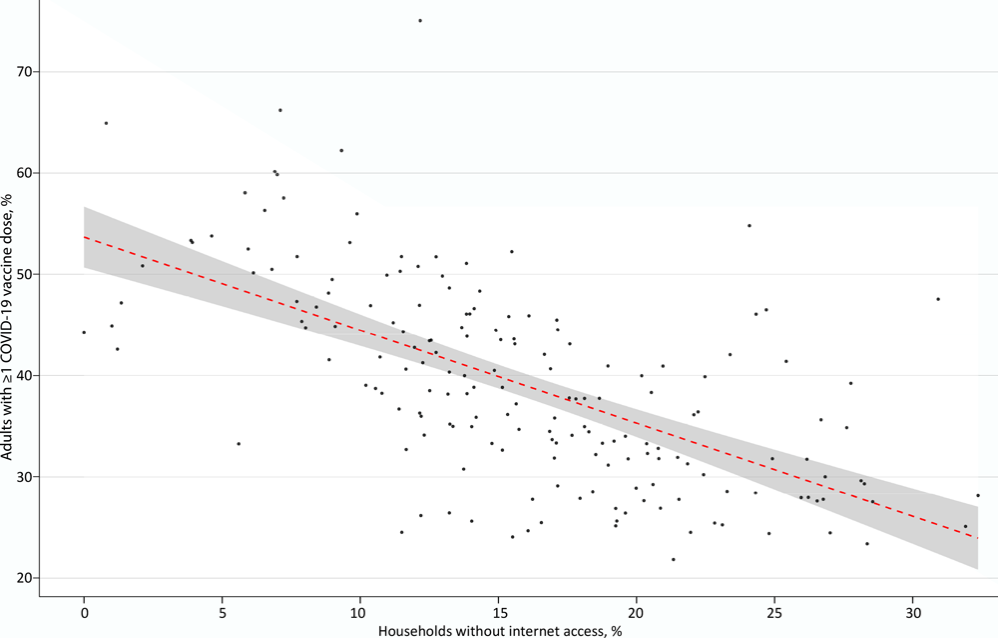
Association in New York City, at the zip code level, between the percentage of households without internet access and the percentage of adult residents with at least 1 COVID-19 vaccine dose. Each point represents 1 zip code. The dashed line represents a simple linear regression model, and the shaded area indicates 95% CIs. Linear regression summary: β = −0.92; 95 CI, −1.09 to −0.75; intercept = 53.7%; *P* < .001; adjusted *R* ^2^ = 0.38. Data sources: New York City Department of Health and Mental Hygiene (8), NYC Open Data (7). Data retrieved on April 7, 2021.

## Action

COVID-19 vaccination was significantly associated with household internet access in New York City at the zip code level. Although this association neither implies nor precludes causation, and does not control for possible confounders, it is consistent with the hypothesis that lack of internet access is a barrier to vaccination. Internet access is a known social determinant of health (15).

Disparities in internet access exist across multiple socioeconomic dimensions and disproportionately affect low-income neighborhoods (16). A digital health divide in the older population has been widely documented (17). A study published in 2021 suggested that the digital health divide is associated with age, education, income, and race/ethnicity (18).

To facilitate equitable and efficient COVID-19 vaccine uptake in New York City, public health officials should establish appointment-free vaccination sites, work with other agencies and organizations to advocate for legal and policy approaches that increase internet access (19), and increase access to in-person and telephone-based services that provide assistance with vaccine appointment scheduling, especially in zip codes with low rates of internet access. Our map can be used to inform the placement of such interventions. Because most disparities in internet access and vaccination were in zip codes in the Bronx and Brooklyn, appropriate partners for public health officials in these areas include the offices of the borough president, the borough-based New York City Department of Health and Mental Hygiene Neighborhood Health Action Centers, and borough-wide community-based organizations. More generally, we recommend that bivariate mapping be considered when selecting methods for comparing geographic distributions of health determinants and health outcomes.

The availability and accessibility of COVID-19 vaccine in New York City has continued to improve. After this writing, home-based vaccination, appointment-free walk-up vaccination sites, mobile vaccination sites, and pop-up vaccination sites were established (20).
